# Supplementary material for: The Evolution and Characterization of the RNA Interference Pathways in Lophotrochozoa
Source: Genome Biol Evol. 2024 May 7;16(5):evae098. doi: 10.1093/gbe/evae098 (PMC11114477; doi:10.1093/gbe/evae098)
Supplement: evae098_Supplementary_Data [file evae098_supplementary_data.zip › Supplementary_materials.zip!/selected_miRNAs/DaRe_selected_miRNas/DaRe_NC_007132.7_35887.pdf]

Provisional ID	:	NC_007132.7_35887
Score total	:	1.7
Score for star read(s)	:	-1.3
Score for read counts	:	0
Score for mfe	:	2
Score for randfold	:	1.6
Score for cons. seed	:	-0.6
Total read count	:	823
Mature read count	:	797
Loop read count	:	0
Star read count	:	26

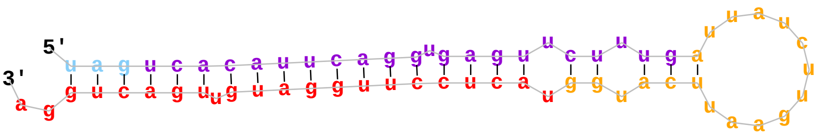

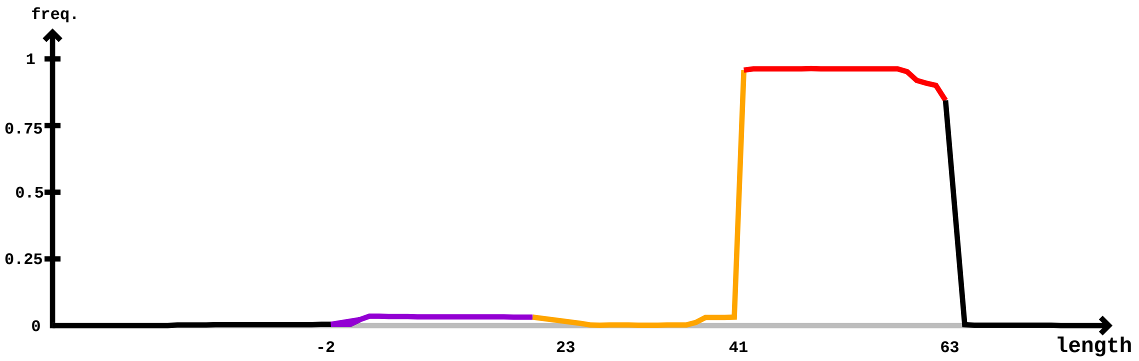

Star Mature

```
5'- ggucaggaauaucucuuugaaugcuucuagucacauucaggugaguucuuugauuaucuugaauucaugguacuccuuggauguugacuggaaguauucuggagcaaa   -3'   obs
    ggucaggaauaucucuuugaaugcuucuagucacauucaggugaguucuuugauuaucuugaauucaugguacuccuuggauguugacuggaaguauucuggagcaaa         exp
    ............((((..(((((((((((((((((((((((.((((.((.(((...........))).)).))))))))))))).))))))))))))))).))))....   reads   mm   sample
    ........................................................ucuugaauucaugguacuccuu..............................   1       0    Gu1
    ..................................................................augguacuccuuggauguugacu...................   2       0    Gu1
    ......................................................................uacuccuuggauguugacugg.................   1       0    Gu1
    ......................................................................uacuccuuggauguugacugga................   16      0    Gu1
    ......................................................................uacuccuuggauguugacuggaC...............   1       1    Gu1

    ...........ucucuuugaaugcuucuagu.............................................................................   1       0    Ov3
    ..............................ucacauucaggugaguucuuug........................................................   4       0    Ov3
    ..............................ucacauucaggugaguucuuuga.......................................................   2       0    Ov3
    ..............................ucacauucaggugaguucuuugauuaucu.................................................   1       0    Ov3
    ...............................cacauucaggugaguucuuuga.......................................................   3       0    Ov3
    ...............................cacauucaggugaguucuuugau......................................................   2       0    Ov3
    ...............................cacauucaggugaguucuuugauG.....................................................   1       1    Ov3
    ..............................................................auucaugguacuccuuggauguugacu...................   1       0    Ov3
    .................................................................caugguacuccuuggauguuga.....................   4       0    Ov3
    ..................................................................augguacuccuuggauguugac....................   1       0    Ov3
    ..................................................................augguacCccuuggauguugacu...................   1       1    Ov3
    ..................................................................augguacuccuuggauguugacu...................   5       0    Ov3
    ......................................................................Cacuccuuggauguugac....................   1       1    Ov3
    ......................................................................uacuccuuggauguugac....................   10      0    Ov3
    ......................................................................uacuccuuggauguugacug..................   6       0    Ov3
    ......................................................................uacuccuuggauguugacugg.................   22      0    Ov3
    ......................................................................uacuccuuggauguugacGgga................   1       1    Ov3
    ......................................................................uGcuccuuggauguugacugga................   1       1    Ov3
    ......................................................................uacuccuuggauguGgacugga................   1       1    Ov3
    ......................................................................uacuccCuggauguugacugga................   1       1    Ov3
    ......................................................................uacuccuuggauCuugacugga................   1       1    Ov3
    ......................................................................uacuccuuggauguugacugga................   285     0    Ov3
    ......................................................................uacuccuuggauguugacuggCa...............   1       1    Ov3
    ......................................................................uacuccuuggauguugacuggUa...............   1       1    Ov3
    ......................................................................uacuccuuggauguugacuggaC...............   36      1    Ov3
    ......................................................................uacuccuuggauguugacuggaa...............   2       0    Ov3
    .......................................................................acuccCuggauguugacuggaa...............   2       1    Ov3
```

```
                              Star                                    Mature
ggucaggaauaucucuuugaaugcuucuagucacauucaggugaguucuuugauuaucuugaauucaugguacuccuuggauguugacuggaaguauucuggagcaaa

..............................ucacauucaggugaguucuuuga.......................................................    1    0    Gu2
......................................................................uacuccuuggauguugacugga................    5    0    Gu2
.............................................................................uggauguugacuggaaguauucuAga.....    1    1    Gu2

..........................cuagucacauucaggGgagu..............................................................    1    1    Ov2
..............................ucacauucaggugaguucuuug........................................................    1    0    Ov2
...............................cacauucaggugaguucuuuga.......................................................    1    0    Ov2
...............................cacauucaggugaguucuuugau......................................................    1    0    Ov2
..................................................................augguacuccuuggauguuga.....................    1    0    Ov2
..................................................................auggCacuccuuggauguugac....................    1    1    Ov2
..................................................................augguacuccuuggauguugac....................    3    0    Ov2
.....................................................................guacuccuuggauguugacugg.................    1    0    Ov2
......................................................................uacuccuuggauguugac....................    5    0    Ov2
......................................................................uacuccuuggaGguugacug..................    1    1    Ov2
......................................................................uacuccuuggauguugacugg.................    12   0    Ov2
......................................................................uacuccCuggauguugacugga................    1    1    Ov2
......................................................................uacuccuuggauguugaAugga................    1    1    Ov2
......................................................................uacuccuuggauguugacuggG................    1    1    Ov2
......................................................................uacuccuuggauguugacugga................    185  0    Ov2
......................................................................uacuccuuggauguugGcugga................    1    1    Ov2
......................................................................uacuccuugUauguugacugga................    1    1    Ov2
......................................................................uacuccuuggauguugacuggU................    1    1    Ov2
......................................................................uacuccuuggauguugacuggaU...............    1    1    Ov2
......................................................................uacuccuuggauguugacuggaC...............    14   1    Ov2
.......................................................................acuccCuggauguugacugga................    1    1    Ov2

......................................................................uacuccuuggauguugac....................    1    0    Gu3
......................................................................uacuccuuggauguugacugga................    5    0    Gu3

...........ucucuuugaaugcuucuaguca...........................................................................    1    0    Ov1
...............uuugaaugcuucuagucacau........................................................................    1    0    Ov1
..............................ucacauucaggugaguucuuug........................................................    3    0    Ov1
..............................ucacauucaggugaguucuuuga.......................................................    3    0    Ov1
...............................cacauucaggugaguucuuug........................................................    1    0    Ov1
...............................cacauucaggugaguucuuuga.......................................................    1    0    Ov1
...............................cacauucaggugaguucuuugau......................................................    2    0    Ov1
.................................................................caugguacuccuuggauguuga.....................    3    0    Ov1
.................................................................caugguacuccuuggauguugaA....................    1    1    Ov1
..................................................................augguacuccuuggauguuga.....................    1    0    Ov1
..................................................................augguacuccuuggauguugac....................    1    0    Ov1
......................................................................uacuccuuggauguugac....................    4    0    Ov1
......................................................................uacuccuuggauguugacugA.................    3    1    Ov1
......................................................................uacuccuuggauguugacugg.................    9    0    Ov1
......................................................................uacuccuuggauguugacugAa................    1    1    Ov1
......................................................................uacuGcuuggauguugacugga................    1    1    Ov1
......................................................................uacuccuuggUuguugacugga................    1    1    Ov1
......................................................................uacuccuuggauCuugacugga................    1    1    Ov1
......................................................................uacuccuuAgauguugacugga................    1    1    Ov1
......................................................................uacuccuuggauguugacuggN................    1    1    Ov1
......................................................................uacuccuuggauguugacugga................    129  0    Ov1
......................................................................uacuccuuggauguAgacugga................    1    1    Ov1
......................................................................uacuccuuggauguugacuggaC...............    17   1    Ov1
......................................................................uacuccuuggauguugacuggaaA..............    1    1    Ov1
.......................................................................acuccCuggauguugacuggaag..............    1    1    Ov1
```
